# What do sales data tell us about implant survival?

**DOI:** 10.1371/journal.pone.0171128

**Published:** 2017-02-21

**Authors:** Rudolf Seemann, Alexander Jirku, Florian Wagner, Arno Wutzl

**Affiliations:** 1 University Clinic of Cranio-, Maxillofacial and Oral Surgery, Medical University of Vienna, Vienna, Austria; 2 Alexas AG, Mauren, Liechtenstein; Virginia Commonwealth University, UNITED STATES

## Abstract

**Objective:**

The aim of this study was to evaluate the influence of implant diameter, length and shape on a surrogate parameter of implant survival; i.e. the implant return rate in a big data analysis.

**Materials and methods:**

A retrospective study was conducted and the factors influencing the success rates of 69,377 sold implants over a seven-year period were evaluated. The osseointegration program of a reseller provides reliable data of a single country. Implant loss rates were investigated using logistic regression models and regressed by implant type, diameter, and length.

**Results:**

The return rate of 69,377 sold implants was 2.78% and comparable to implant loss rates in previous published prospective studies as its surrogate parameter. A total of 80% of implant returns had occurred within 157 days, and an additional 15% within 750.25 days. Diameters of 3.8 to 5.0mm showed the lowest return rates with its bottom in the 4.3mm implant whilst 6.0mm implants had significantly higher return rates. In comparison to the most sold implant length (13mm) shorter implants showed significantly higher early return rates.

**Conclusions:**

The study provides evidence that in cases of standard indications and sufficient bone, the use of screw typed dental implants with 3.8 or 4.3 diameter and 11 or 13 mm length shows the lowest implant return rates. Other implants may be selected only in specific indications.

## Introduction

The history of dental implants shows a steady increase in cumulative survival rates (CSR). In the nineties a successful implant was defined as one with a 5-year CSR of 89.9% [1]. However, dental implantology has currently achieved a 5-year CSR of 97.2% (95% CI 96.3–97.9%) in implant-supported single crowns [2], and 95.6% in implant-supported fixed dental prostheses [3]. Although the quest for higher success rates in the last few years has led to no more than the production of a titanium screw, it has been substantially improved by subtle modification of its microgeometry and nanogeometry. The platform switch reduces marginal bone loss compared to platform match abutments [4] without improving the implant success rate [5]. In addition to being visible to the naked eye, the properties of the implant have been altered on the microscale and even nanoscale level. A number of surface treatments including sandblasting, etching, coating, UV irradiation, helium irradiation, and ion implantation have improved osseointegration and enhanced success rates [6].

Implant length and implant diameter seem to influence the long-term outcome of implant treatment. As shown in a recent review, the survival rate of small-diameter implants appears to be similar of that of implants with a regular diameter [7]. Moreover, short implants seem to be associated with the same outcome as long as the diameter is regular and not reduced. Since grafting procedures or a clinical situation calling for grafting influence implant survival negatively, implant success rates are about 92% after sinus augmentation procedures and 93% in grafted areas [8].

**Fig 1:**
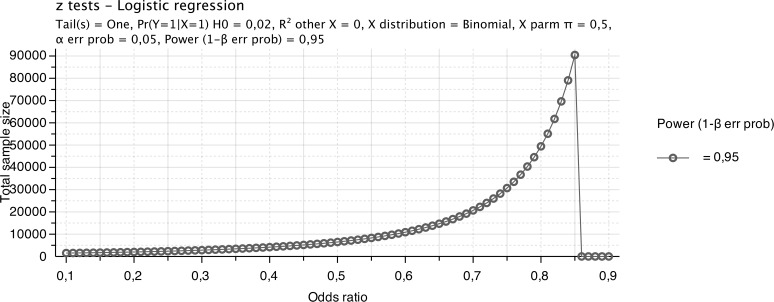
Power analysis of implant failure. Estimated sample size of a logistic regression model depending on a varying odds ratio with a significance level of 5%, a power of 95%.

The high technological turnover calls for studies based on short-term models. Scientists are no longer combating the last two to three percent of failure, but are focused on marginal bone loss and esthetics. Sample sizes measured in multiples of ten thousands would be needed to overcome the last three percent (Fig 1). However, do we have surrogate parameters of success that can be observed on a larger scale?

In the present study we analyze implant success from the industrial perspective. Implant failure results in the returned implant being declared a failure by the dental surgeon or the dentist. One reseller provided the data for the present investigation. Its main objective was to evaluate the influence of diameter, length, surface, area and surface type on early implant return, based on sales and return report data.

## Materials and methods

We evaluated the factors influencing the success rates of the implants provided by one reseller (Altatec GmbH, Wimsheim, Germany) over a seven-year period (2007–2013) in one country (Austria).

The fact that only sales data were used in this study and no patients were included rendered the ethical committee vote unnecessary. All implants inserted in the preceding years and returned during the study period were included in the study. The *implant return ratio*, defined as the number of returned implants divided by sold implants of a certain length, diameter, and type during the study period, was the principal surrogate parameter of clinical implant success. Loss data were collected by one Austrian reseller (Alltec Dental GmbH, Dornbirn, Austria) as part of their *10-year Osseointegration Guarantee Program*. Losses were reported with regard to implant level, type, diameter, length, time from implantation to failure, and the presumed reason for failure. Subdividing time to failure into three categories yielded an economic model of loss: type A (80% early losses), type B (15% delayed losses), and type C (5% late losses). Other researchers can access the sales data by contacting the corresponding author.

The numbers of sold implants per year were provided in tabular form, stratified by implant type (Camlog® Root-Line, Screw-Line Promote®, Screw-Line Promote Plus®), length (9, 11, 13, 16 mm), diameter (3.8, 4.3, 5.0, 6.0 mm), and year. All implants have the sandblasted and etched Promote ® surface. Subtle differences exist between the three implant types. The Root-line implant (RL) is a root-shaped implant with a 2-mm-wide machined neck. The Screw-line implant is non-tapered and provides less apical freedom. The Screw-Line Promote® (SLP) has a 1.4-mm machined implant neck, and the Screw-line Promote® plus (SLPp) a 0.4-mm machined neck.

### Austrian demography and the density of dentists

In 2006 a total of 8,254,298 individuals (4,014,344 men and 4,239,954 women), of whom 7,457,632 were Austrian citizens and 796,666 non-Austrian, were residing in Austria (source: Statistik Austria, Vienna). At the last microcensus 2006/2007, 27.5% of men and 19.4% of women reported to be smokers (source: Statistik Austria, Vienna); 5.4% of all men and 15.9% of men older than 60 years, 6.4% of all women and 19.0% of women older than 60 years had diabetes. In all 4,451 dentists were registered at the Austrian Chamber of Dentists (Österreichische Zahnärztekammer, Vienna, Austria) in 2006. Since no further specialization is needed, all dentists are authorized by law to insert implants. Besides, according to the Austrian Society of Oral and Maxillofacial Society (Österreichische Gesellschaft für Mund-, Kiefer- und Gesichtschirurgie, Vienna, Austria), 13 maxillofacial clinics exist in Austria.

### Statistical analysis

Three logistic regression models were computed: one for early/type A loss (80% of all losses occurred within 160 days after implantation), one for delayed/type B loss (15% of all losses occurred between 160.1 and 761.4 days after implantation), and one for late/type C loss (5% of all losses occur at least 761.5 days after implantation). Loss was regressed by implant type, diameter, and length. Univariate, i.e. crude odds ratios (ORs), multivariate (i.e. adjusted) ORs and 95% confidence intervals (CI) of the ORs were provided. Wald’s test (probability of OR equal to 1, i.e. parameter has no influence) and the likelihood ratio test (likelihood ratio test of model reduced by the factor compared to the full model) were calculated. The most frequently sold implant (Screw-Line Promote®, 4.3 mm in diameter and 13 mm in length) was taken as reference.

## Results

A total of 69,377 sold implants were included in this study. Of these, 11,220 were RL implants, 32,775 SLP, and 25,382 SLPp. Of the 69,377 implants, 2.78% (n = 1,928) were returned due to loss. Of the returned implants 225 were excluded due to missing information: 182 multiple losses with ambiguous mention of diameter and length, and 43 due to missing implantation or explantation date. The excluded implants accounted for nearly equal fractions in the implant lines: 0.39% of Root-Line implants, 0.36% of Screw-Line implants, and 0.24% of Screw-Plus implants. All 1703 implant returns, accounting for 88.3% of all returns, qualified for the *10-Year Osseointegration Guarantee Program* and were finally included in the analysis. Sales data were corrected to reflect the true implant return rate of 2.78% by scaling the sold number of implants with a correction factor resembling the fraction of included returns (Root 0.895, Screw 0.847, Screw+ 0.893).

The median overall implant return rate (IRR) was 3.01%, and the interquartile range (IQR) 2.17%. The median type A IRR was 2.47% (IQR: 1.76%), ranging from 0.56% for the 5x16 mm Screw-Plus (n = 179) to 12.0% for the 6x9 mm Root-Line implants (n = 25). The median type B IRR was 0.37% (IQR 0.61%), ranging from 0.0% for 13 different implant types (RL 4.3x16, 5x15, SLP 4.3x16, 5x16, 6x9, 6x13, SLPp 4.3x16, 5x16, 6x9, 6x11, 6x13, 6x16) to 6.67% for the 6x16 mm RL. The median type C IRR was 0.03% (IQR 0.19%), ranging from 0.0% for one half of the 48 implant types (3 types x 4 diameters x 4 lengths) to 4.0% for the 6x9 RL.

**Fig 2:**
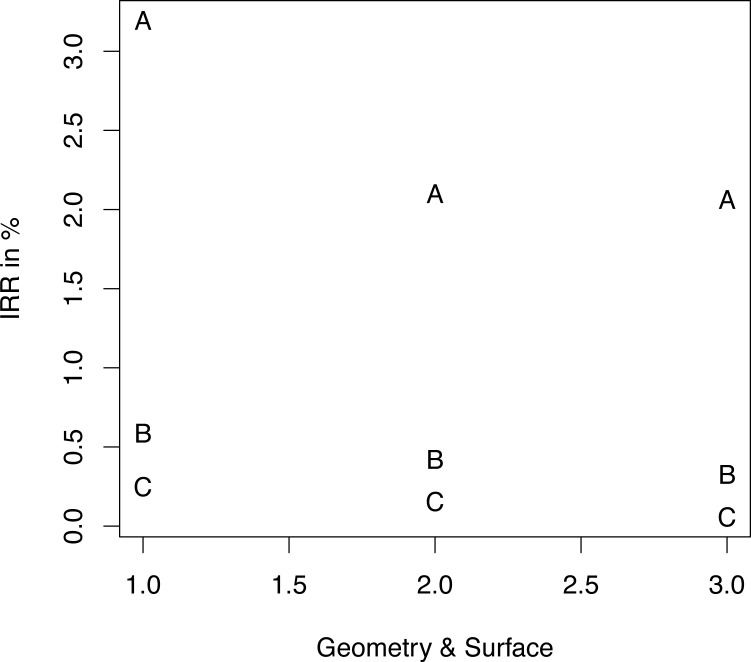
Implant return ratio versus type. Implant return ratio (IRR) of early failure (A), intermediate failure (B) and late failure (C) of root-shaped implants (1), screw-shaped implants with polished neck (2), and screw-shaped implants with reduced polished neck (3).

**Fig 3:**
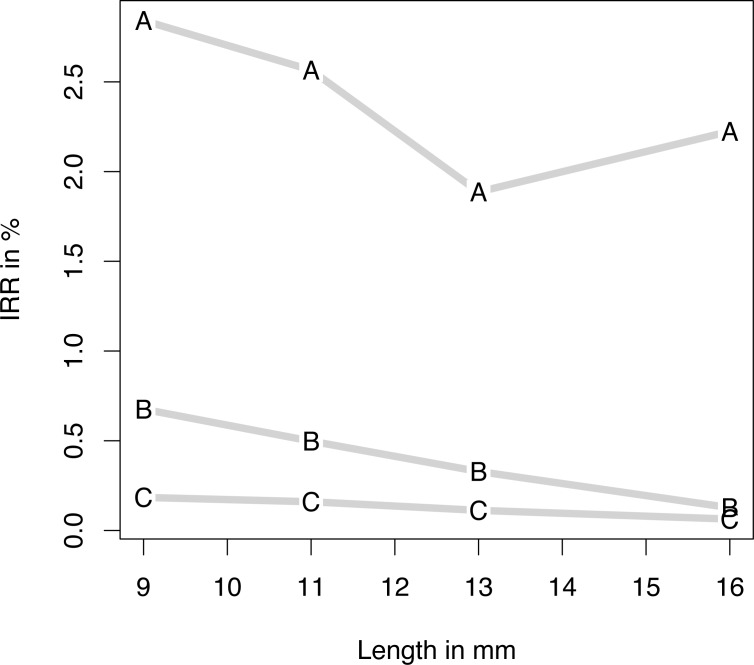
Implant return ratio versus length. Implant return ratio (IRR) of early (A), intermediate (B), and late (C) failure of four lengths: 9, 11, 13, and 16 mm.

**Fig 4:**
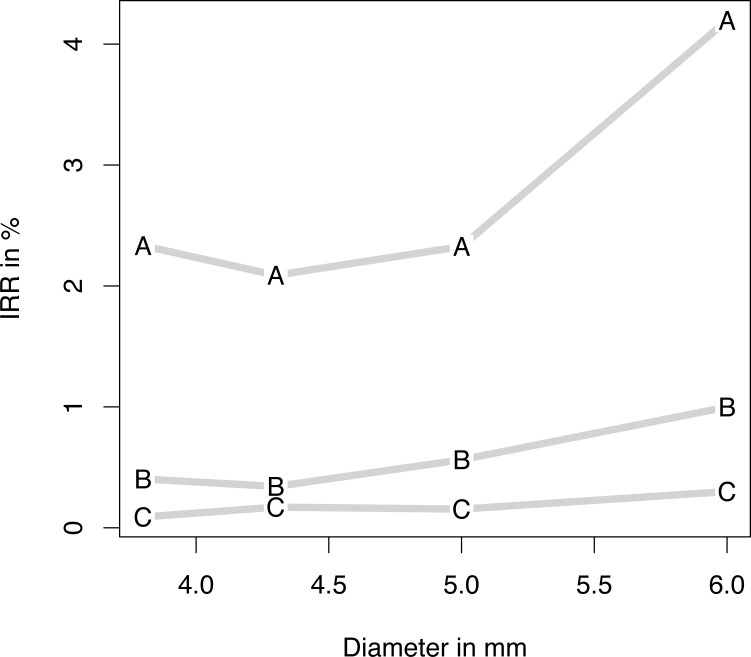
Implant return ratio versus diameter. Implant return ratio (IRR) of early (A), intermediate (B), and late (C) failure of four diameters: 3.8, 4.3, 5, and 6 mm.

Loss occurred within a median period of 69 days (IQR 98 days; 90% CI 7.75–750.25 days) after implantation (maximum 3564 days). Eighty implant losses were attributed to implants inserted between 2001 and 2006 (2001–2006: 1; 2; 4; 5; 16; 52). Three loss categories were defined according to the ABC rule: 80% of implant returns had occurred within 157 days, and an additional 15% within 750.25 days. The remaining 5% were classified as type C/late loss. Early (type A), delayed (type B) and late (type C) implant return rates were compared with implant-specific parameters: geometry (Fig 2), length (Fig 3), and diameter (Fig 4).

*Logistic regression models* of type A, B, and C losses are given in Table 1. With regard to the geometry and surface, in all loss categories RL achieved significantly higher loss rates than did SLP. SLPp showed significantly lower late loss rates than did SLP. Apart from statistically equal return rates for 13- and 16-mm implants, the shorter the implant was, the higher the type A losses were. Delayed and late losses showed a strictly linear dependence. While higher return rates were registered for implants thinner or thicker than 4.3 mm in both type A and type B returns, a linear dependence of late losses was noted; the smaller the diameter, the fewer were the returns.

**Table 1:** Logistic regression of implant return vs. type, length and diameter. Logistic regression models of Early/ Type A loss (80% of all losses occurred within 160 days after implantation), Delayed / Type B loss (15% of all losses occurred between 160 and 761.5 days after implantation), and Late / Type C loss (5% of all losses occur at last 761.5 days after implantation). Crude odds ratios (OR) are univariate, adjusted OR are multivariate models. In round brackets the 95% confidence interval (CI) of the OR is printed. Probability of the Wald’s test (probability of OR equal to 1, i.e. parameter has no influence) and likelihood ratio test (likelihood ratio test of model reduced by the factor compared to full model). The most sold implant (Screwline with 4.3 mm diameter and 13 mm length) was taken as reference.

		Type: ref. = Screw		Length: ref. = 13			Diameter: ref. = 4.3		
		Root	Screw^+^	9	11	16	3.8	5.0	6.0
**Type A Loss**	crude OR	1.52	0.98	1.51	1.36	1.18	1.12	1.11	2.01
	adj. OR	1.6	0.97	1.59	1.41	1.08	1.14	1.05	1.77
	P_Wald's test_	< 0.001	0.598	< 0.001	< 0.001	0.481	0.037	0.598	< 0.001
	P_LR-test_	< 0.001		< 0.001			< 0.001	
**Type B Loss**	crude OR	1.39	0.77	2.06	1.51	0.39	1.18	1.65	2.92
	adj. OR	1.57	0.75	2.17	1.57	0.35	1.23	1.5	2.42
	P_Wald's test_	0.006	0.056	< 0.001	0.002	0.013	0.168	0.024	0.009
	P_LR-test_	< 0.001		< 0.001			< 0.001		
**Type C Loss**	crude OR	1.61	0.37	1.64	1.43	0.57	0.52	0.9	1.75
	adj. OR	1.78	0.36	1.9	1.55	0.5	0.54	0.8	1.39
	P_Wald's test_	0.025	0.001	0.065	0.077	0.253	0.018	0.479	0.582
	P_LR-test_	< 0.001		< 0.001			< 0.001		

### Reasons for failure

Reasons for implant loss could be mentioned optionally for each returned implant. In type A losses, 57.5% were classified as osseointegration failures while no reason was provided in 28.1% of cases. Infections were the third most common reason (9.7%), followed by biomechanical causes such as early/immediate loading or dentures riding on healing caps, insufficient primary stability and bruxism in 2.4%. Misplaced implants (too close to the inferior alveolar nerve or adjacent teeth or neighboring implants) were reported for 0.5% of returns. Delayed—type B–losses were not commented in 45.8% while 36.4% were rated as failed osseointegration, followed by periimplantitis in 6.8%, inflammation in 3.8%, augmentation in 3.4%, low primary stability in 1.7%, insufficient bone in 0.8%, bruxism in 0.8%, and dentures in 0.4%. The late–type C–implant failure was not classified in 47.4%, was rated as missed osseointegration in 23.1%, periimplantitis in 19.2%, inflammation in 5.1%, insufficient bone in 2.6%, augmentation in 1.3%, and low primary stability in 1.3%.

## Discussion

A total of 69,377 sold implants were included in this study. Compared to other studies, this is the highest ever published number of implants that have been followed up. With regard to primary implant failure (type A–within the first 157 days), the implant with the highest probability of success (no return) was the 4.3 x 13 mm screw-type implant.

Of 69,377 implants, 2.78% (n = 1,928) had been returned due to loss. This number is similar to previously published cohorts. The 5-year implant survival rate was estimated at 97.7% and, based on four prospective studies including 124 implants, the 10-year implant survival rate was estimated at 94.9% [9]. Of the 4266 inserted implants, 216 were lost. 47% (102/216) of them were lost before loading and 53% (114/216) after loading [10] The estimated yearly failure rate ranged from 0 to 2.94. The estimated 5-year survival rate is 95.6%.

Instead of performing a prospective study we used the implant return rate reported in the *10-Year Osseointegration Guarantee Program* for the surgeon/dentist and patients. Being identified as such by the manufacturer, the data may have been subject to bias. In contrast to a clinical study with selected surgeons, our sales data reflect a cross section of all dentists and surgeons using these implants. Implants in this study were either placed by generalists or maxillofacial surgeons. There is no specialty on oral surgery in Austria. Of all losses 31.1% were placed by maxillofacial surgeons. All other failed implants were returned by dentists holding a postgraduate diploma in implantology. No data was provided on working experience of the surgeons. In a recent study, Jemt et al. evaluated whether the factor surgeon played a significant role in the frequency of implant failures of 39,961 dental implants placed by 23 surgeons over 28 years. While significant differences in implant failure between surgeons were found, the experience did not play a significant role. Although surgeons reduced their overall failure rate when the protocol was changed from turned to moderately rough implants they interestingly maintained their relative level of failure rate. Female surgeons were found to have significantly lower failure rates when implants with turned surfaces were inserted which was attributed to potential gender differences in risk taking.[11]

The typical scientific perspective is characteristically the skilled surgeon’s point of view. From the patient’s point of view, different conventional dentists inserting implants may signify different probabilities of failure. These results may be indicative of the fact that, under specific conditions, certain types of implants have to be inserted by skilled surgeons.

A potential limitation of this study is that surgeons may not return all failed implants or not right after failure. Accidentally opened implants might be returned and declared as implant failure. Although surgeons may have different preferences and stocks of implant sizes, the implant return rate is the quotient of the total number of returned implants divided by the number of sold implants for each category. Nevertheless, our sales data correlate with the data of previously outcome studies. The large number of investigated implants permits us to draw conclusions about the investigated parameters. A statistical analysis of a 2% percent failure rate needs a cohort of nearly 70,000 implants to draw serious conclusions (Fig 1).

Another limitation is given by the geographic uniqueness of the study including Austrian patients, which may limit generalization to other population. Especially smoking considerably affects implant survival (OR = 1.96).[12] On the other hand smoking habits vary considerably between countries. In Austria figures on smoking are provided by Statistics Austria and constitute 24.3% of the Austrian population with an increasing percentage of smoking women (22.1% vs. 26.5% men in 2014). In comparison to 18% prevalence of smoking in the world’s population.[13]

Are implant failures predictable? Each implant has a certain likelihood of success or failure. The failure possibility of a 5x16mm implant is 0.56% while that of a 6x9mm implant is 12% in group A, or 6% in group B. These figures are not related to the indication. The indications for shorter implants differ from those for longer implants. The indication for a smaller diameter also differs from that for a larger diameter. The surgeon has to keep the possibilities of failure in mind.

SLPp showed significantly lower late loss rates than did the same implant with a Promote® surface. The former have a rough surface to the neck, sparing just the last 0.4 mm. The latter have a machined 1.4-milimeter-wide neck. The microstructure of the SLPp implants may enhance bone and soft tissue intergration [14]. Interestingly, the root implant is associated with more losses than the straight implants. Recently, rough and microthreaded neck implants were proven to show less marginal bone loss than machined-neck implants. [15]

Besides statistically equivalent return rates for the 13- and 16-mm implants, the shorter the implant was, the higher the type A losses were. We believe that the risk of implant loss is higher in shorter implants because of reduced primary stability. In a meta-analysis of randomized controlled trials, Lee et al. investigated the use of 5-mm to 8-mm implants without augmentation versus implants < 8 mm with augmentation in the posterior region, where the vertical height of bone was limited [16]. The authors registered no difference, but it should be noted that they only took well defined RCT into account; these do not necessarily reflect the actual situation because patients are selectively included in the studies.

The results of 4591 implants inserted at a single private medical office over a 10-year period were investigated in another recent study [17]. In a multivariate analysis, the authors identified several risk indicators for failure: implant location, length, design, timing of implantation, bone grafting procedures and gender. The length of the implant was identified as a risk factor: short implants placed in the maxilla showed a higher risk of failure than did standard or longer implants.

In logistic regression models we identified several risks factors. Shorter implants were subject to a greater risk of failure than longer implants. However, the indications and the situation prior to the indication were not determined. Therefore, the reason for implant loss may have been the compromised bone situation rather than the short implant.

Higher failure rates with short implants [18] have to be expected. However, the surface characteristics of the implants were not reported. Shorter implants with reduced diameters are prone to a higher failure rate than longer implants with reduced diameters [7, 19]. In contrast, implant length with regular diameters has no significant impact on the survival rate of dental implants [20]. In 2010 Romeo et al. concluded that there is no statistically significant difference in the survival of shorter implants compared to implants of standard dimensions [21]. These conflicting data could possibly be attributed to primary stability, the patients´ bone quality, and the surgeons’ learning curve. Another important point is the fact that longer implants leave larger bone defects after implant loss because of periimplantitis. Revision surgery and the insertion of a second implant may be impossible after a failed long implant, but not after loss of a short and thin implant.

Delayed and late losses showed a strictly linear dependence. While higher return rates were registered for implants thinner or thicker than 4.3 mm in both type A and type B returns, a linear dependence of late losses was noted: the smaller the diameter, the fewer the returns. The survival rate of 98.7% after 5 years was reported for patients with narrow-diameter implants [22]. Significantly more implant failures were registered using a wide platform (5 mm) than a regular one (4 or 3.75 mm) [8]. Given the fact that success rates were not influenced by diameter in other studies [23, 24] we believe that the apparent negative effects of implant diameter were influenced by the decision to use wider implants in areas that could not accommodate longer implants. Implants with reduced diameters exhibit the same survival rates (95–100%) as do implants with wider diameters [7].

The main advantage of small-diameter implants is that the surgeon can perform less invasive surgical procedures in patients with compromised bone and those with a reduced interradicular space. Our results are consistent with those reported in other studies.

Eighty percent of implant returns had occurred within 157 days. Several authors have distinguished between early and the late failure. Early failures are related to the patient/indication/surgeon’s experience, whereas late failures are attributed to prosthetic conditions such as overload of the prosthesis or bone loss [25].

Our data were based on the sales figures and implant return rates registered during a reseller’s 10-year guarantee program, and were focused on the gross structure and microstructure of the dental implants. The strength of the investigation is the fact that a very large number of implants was studied. The data registered in the study are valuable for a medical team when selecting an implant type for a specific patient. The superiority of one implant over another for specific indications should be investigated further in a randomized clinical trial. Another strength of the present study is that it was not influenced by the patients’ health conditions, surgical indications, or risk factors such as smoking or type of bone.

This study reports the highest number of implants ever studied in a single investigation. Length and diameter have a significant impact on implant return rates. Sales data may predict the likelihood of implant success. The study shows that, given standard indications and sufficient bone, the general surgeon may use the universal SLPp and SLP implants of 3.8 or 4.3 diameter and 11 or 13 mm length. The other implants described in this report may be selected in the presence of specific indications and for specific planned treatments.

## Supporting information

S1 File Loss and sales data. This excel file contains loss and sales data. Type of implant (root, screw, screw_plus) is provided in column 1, length of sold implant in column 2, diameter in column 3, respectively, Type A (early losses) in column 4, type B (intermediate) losses in column 5, type C (late) losses in column 6, total number of not returned implants in column 7. Thus, the total number of sold implants of one type, length and diameter is the sum of colmns 5, 6, and 7.
(XLSX)
